# Copper Resistance Promotes Fitness of Methicillin-Resistant Staphylococcus aureus during Urinary Tract Infection

**DOI:** 10.1128/mBio.02038-21

**Published:** 2021-09-07

**Authors:** Panatda Saenkham-Huntsinger, Amanda N. Hyre, Braden S. Hanson, George L. Donati, L. Garry Adams, Chanelle Ryan, Alejandra Londoño, Ahmed M. Moustafa, Paul J. Planet, Sargurunathan Subashchandrabose

**Affiliations:** a Department of Veterinary Pathobiology, College of Veterinary Medicine and Biomedical Sciences, Texas A&M University, College Station, Texas, USA; b Department of Microbiology and Immunology, Wake Forest School of Medicine, Winston-Salem, North Carolina, USA; c Department of Chemistry, Wake Forest University, Winston-Salem, North Carolina, USA; d Division of Pediatric Infectious Diseases, Children’s Hospital of Philadelphia, Philadelphia, Pennsylvania, USA; e Department of Pediatrics, Columbia University, New York, New York, USA; f Division of Gastroenterology, Hepatology, and Nutrition, Children’s Hospital of Philadelphia, Philadelphia, Pennsylvania, USA; g Department of Pediatrics, Perelman College of Medicine, University of Pennsylvania, Philadelphia, Pennsylvania, USA; h Sackler Institute for Comparative Genomics, American Museum of Natural History, New York, New York, USA; The Ohio State University School of Medicine

**Keywords:** UTI, copper, *S. aureus*, MRSA, Gram-positive UTI, mouse model

## Abstract

Urinary tract infection (UTI) is one of the most common infectious conditions affecting people in the United States and around the world. Our knowledge of the host-pathogen interaction during UTI caused by Gram-positive bacterial uropathogens is limited compared to that for Gram-negative pathogens. Here, we investigated whether copper and the primary copper-containing protein, ceruloplasmin, are mobilized to urine during naturally occurring UTI caused by Gram-positive uropathogens in patients. Next, we probed the role of copper resistance in the fitness of methicillin-resistant Staphylococcus aureus (MRSA) during experimental UTI in a murine model. Our findings demonstrate that urinary copper and ceruloplasmin content are elevated during UTI caused by Enterococcus faecalis, S. aureus, S. epidermidis, and *S. saprophyticus*. MRSA strains successfully colonize the urinary tract of female CBA mice with selective induction of inflammation in the kidneys but not the bladder. MRSA mutants lacking CopL, a copper-binding cell surface lipoprotein, and the ACME genomic region containing *copL*, exhibit decreased fitness in the mouse urinary tract compared to parental strains. Copper sensitivity assays, cell-associated copper and iron content, and bioavailability of iron during copper stress demonstrate that homeostasis of copper and iron is interlinked in S. aureus. Importantly, relative fitness of the MRSA mutant lacking the ACME region is further decreased in mice that receive supplemental copper compared to the parental strain. In summary, copper is mobilized to the urinary tract during UTI caused by Gram-positive pathogens, and copper resistance is a fitness factor for MRSA during UTI.

## INTRODUCTION

Bacterial colonization of the urinary tract leading to inflammation is among the most common infectious conditions affecting people throughout the world (1, 2). Urinary tract infection (UTI) results in around 11 million physician visits, 1.7 million emergency room visits, and 470,000 hospitalizations, with an annual direct cost of ∼$3.5 billion in the United States (3–6). Inflammation caused by infection of the urinary bladder (cystitis) is the most common clinical presentation of UTI (7). Less common but more serious outcomes of UTI include kidney infection (pyelonephritis), bacteremia, and sepsis (1, 7). Women are four times more likely to develop UTI than men due to anatomic differences. Factors that increase the risk for UTI include age (children and the elderly), catheter use, anatomic/physiologic abnormalities of the urinary tract, diabetes mellitus, or urolithiasis (1, 8, 9). Recurrent episodes of UTI are also common among the high-risk groups, and recurrent UTI is often recalcitrant to treatment with antibiotics (1). Uropathogenic Escherichia coli (UPEC) is the predominant etiological agent of UTI (2, 10). Other clinically significant causes of UTI include Enterococcus faecalis, Klebsiella pneumoniae, Proteus mirabilis, and staphyloccocci (2, 7).

Staphylococcus aureus is a clinically significant causative agent of UTI in individuals with urinary catheter use and also in otherwise healthy individuals, albeit less frequently (2, 11, 12). Additionally, S. aureus is a clinically significant cause of renal abscesses of hematogenous origin (13). S. epidermidis colonization in the urinary tract is a major concern in device-associated infections, including in urinary catheters, with sporadic cases in patients without instrumentation in the urinary tract (14, 15). *S. saprophyticus* is a more frequent cause of UTI in otherwise healthy young women (7, 16). Antibiotic-resistant isolates, particularly methicillin-resistant S. aureus (MRSA), are widely disseminated and are common causes of community-acquired MRSA infections, including UTI (11, 17–22). However, our knowledge on the pathogenesis and host response during UTI caused by staphylococci is limited (16, 23). Here, we investigate the contribution of copper resistance in the fitness of MRSA in a murine model of ascending UTI. Copper has been implicated as an effector of innate defense against a number of bacterial pathogens, and copper contributes to the antibacterial killing mechanism within murine macrophages (24). Chromosomally encoded *copA* and *copZ* genes encode a copper efflux P-type ATPase and a copper metallochaperone, respectively, that are upregulated during copper stress in S. aureus (25–27). These genes facilitate maintenance of low levels of bioavailable copper in the cytoplasm of all S. aureus strains. Some MRSA isolates encode an additional copper efflux P-type ATPase (CopB) and a multicopper oxidase (Mco) on a plasmid that is also capable of integrating into the chromosome (28, 29). Isolates belonging to the USA300 epidemic clone of MRSA are known to carry an additional copper efflux P-type ATPase (CopX) and an extracellular copper-binding lipoprotein (CopL) (20, 21, 30, 31). Genes encoding CopX and CopL are found in both the ACME (arginine catabolic mobile element) genomic region in the North American USA300 isolates and the COMER (copper and mercury resistance) genomic region in the South American USA300 isolates.

Here, we present findings from our investigation using human clinical samples, *in vitro* assays, and a mouse model of UTI on the role of copper at the host-pathogen interface during UTI caused by S. aureus. We evaluated whether copper and ceruloplasmin, a copper-containing protein, are mobilized to urine in patients with UTI caused by S. aureus and other Gram-positive pathogens. We used MRSA isolates of the USA300 lineage in this study because they exhibit higher levels of resistance to copper than S. aureus isolates from other lineages (21, 30, 31). Copper resistance phenotypes of mutants lacking ACME, CopL, and CopX were validated *in vitro*, and their role in fitness of S. aureus was interrogated in a murine model of UTI. Our findings also indicate a novel link between copper toxicity and iron homeostasis in S. aureus. Collectively, our findings paint a portrait in which copper is mobilized to urine during UTI, and copper resistance conferred by ACME and CopL contributes to optimal fitness of S. aureus in a murine model of UTI.

## RESULTS

### Copper and ceruloplasmin are mobilized to urine during UTI caused by Gram-positive bacterial pathogens.

**FIG 1:**
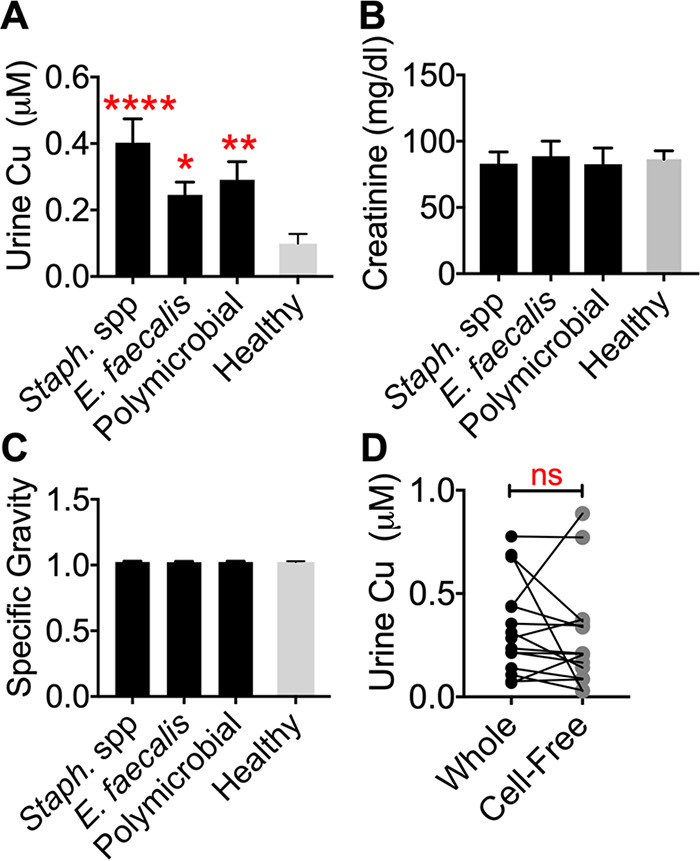
Urinary copper content in human UTI urine samples. (A) Copper content was determined by ICP-MS. Staphylococcus spp., *n* = 20; E. faecalis, *n* = 20; polymicrobial, *n* = 12; healthy controls, *n* = 10. Species are listed in Materials and Methods. *, *P < *0.05; **, *P < *0.01; ***, *P < *0.001, all by analysis of variance (ANOVA) with Dunnett’s posttest. (B) Urinary creatinine content was quantified with a colorimetric assay. (C) Refractometry measurements of specific gravity of urine samples. (D) Copper content of whole and cell-free UTI urine samples (*n* = 15, connected by lines) was determined by ICP-MS. Mean and standard errors of the means (SEM) are reported.

Since copper is involved in protection from multiple bacterial pathogens (24), including UPEC (32–34), we investigated whether urinary copper levels change during UTI caused by Gram-positive pathogens. Urine samples from healthy volunteers and from patients suspected to have UTI based on clinical findings that was verified by laboratory findings were used in this study. Copper content of human urine samples was determined by inductively coupled plasma mass spectrometry (ICP-MS). Statistically significant increases in urinary copper accumulation were noted in UTI urine samples containing Gram-positive pathogens, including S. aureus, S. epidermidis, *S. saprophyticus*, other coagulase-negative staphylococci (listed in Materials and Methods), and E. faecalis (Fig. 1A). Urine samples that were culture positive for two or more bacteria, indicative of polymicrobial UTI, also contained elevated copper levels compared to controls (Fig. 1A). Urinary creatinine content and specific gravity were comparable, indicating that increase in copper content is not due to changes in urine volume or concentration between healthy and UTI groups (Fig. 1B and C). Since complete urine samples were used for ICP-MS analysis, we also checked if increased copper is found in cell-associated or cell-free fractions (Fig. 1D). This is important because large numbers of exfoliated epithelial cells and neutrophils are found in UTI urine samples. We did not observe a difference in copper content in clarified urine compared to complete urine samples, indicating that the elevated level of copper is found in the cell-free fraction of urine during UTI.

**FIG 2:**
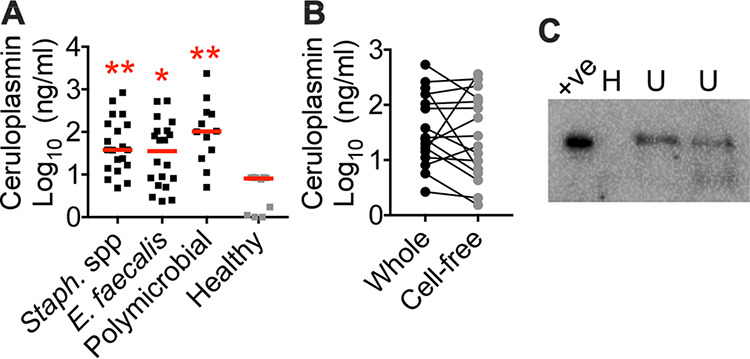
Detection and quantification of ceruloplasmin in urine. (A) ELISA-based determination of urinary ceruloplasmin levels. Staphylococcus spp., *n* = 20; E. faecalis, *n* = 20; polymicrobial, *n* = 12; healthy controls, *n* = 10. Species are listed in Materials and Methods. *, *P < *0.05; **, *P < *0.01, both by Kruskal-Wallis test with Dunn’s posttest. Bars indicate medians. (B) Ceruloplasmin levels in whole and cell-free UTI urine samples (*n* = 15, connected by lines) were also determined by ELISA. (C) A representative immunoblot of healthy (H) and UTI (U) urine samples. +ve, human ceruloplasmin (20 ng).

Ceruloplasmin is a copper-containing protein secreted by the hepatocytes that contains ∼95% of circulating copper in mammals (35). We determined urinary ceruloplasmin levels by enzyme-linked immunosorbent assay (ELISA) to check if this protein is the molecular source of copper during UTI. Urine samples from UTI patients revealed increased levels of ceruloplasmin compared to controls (Fig. 2A). There was no significant difference in the levels of ceruloplasmin in cell-free urine compared to complete urine samples (Fig. 2B). Western blotting was performed to confirm these findings, and ceruloplasmin was detected in UTI urine samples (Fig. 2C). Since copper ions are tightly bound to ceruloplasmin, we tested whether copper could be released from ceruloplasmin by MRSA. Coincubation of ceruloplasmin with USA300 MRSA strain SA116 (Table 1) led to its degradation (see Fig. S1A in the supplemental material). Culture supernatants from MRSA in stationary phase also exhibited a strong ceruloplasmin-degrading activity, as determined by immunoblotting (Fig. S1A). We detected an increase in the abundance of *copA* transcripts, a copper-regulated gene, in MRSA exposed to ceruloplasmin (Fig. S1B). Collectively, our results indicate that copper and ceruloplasmin, a copper-containing acute-phase reactant, were mobilized to urine in humans with naturally occurring UTI caused by Gram-positive uropathogens.

**TABLE 1:** Bacterial strains

Strain	Description	Source or reference
MRSA		
SA116	FPR3757, wild-type MRSA, USA300	71
SA116 *ΔcopX*	Mutant lacking *copX*	30
SA116 *ΔcopX R*	Mutant lacking *copX*, rifampin resistant	This study
SA116 *ΔcopL*	Mutant lacking *copL*	30
SA116 *ΔcopL R*	Mutant lacking *copL*, rifampin resistant	This study
SF8300	Wild-type MRSA, USA300	50
SF8300 *ΔACME*	Mutant lacking ACME	50
SF8300 *ΔACME R*	Mutant lacking ACME, rifampin resistant	This study
UPEC		
CFT073	Wild type	72

10.1128/mBio.02038-21.1 FIG S1 MRSA-ceruloplasmin interaction assays. Download FIG S1, DOCX file, 0.3 MB. Copyright © 2021 Saenkham-Huntsinger et al. 2021 Saenkham-Huntsinger et al. https://creativecommons.org/licenses/by/4.0/ This content is distributed under the terms of the Creative Commons Attribution 4.0 International license.

### Accessory copper resistance genes in urinary S. aureus isolates.

**FIG 3:**
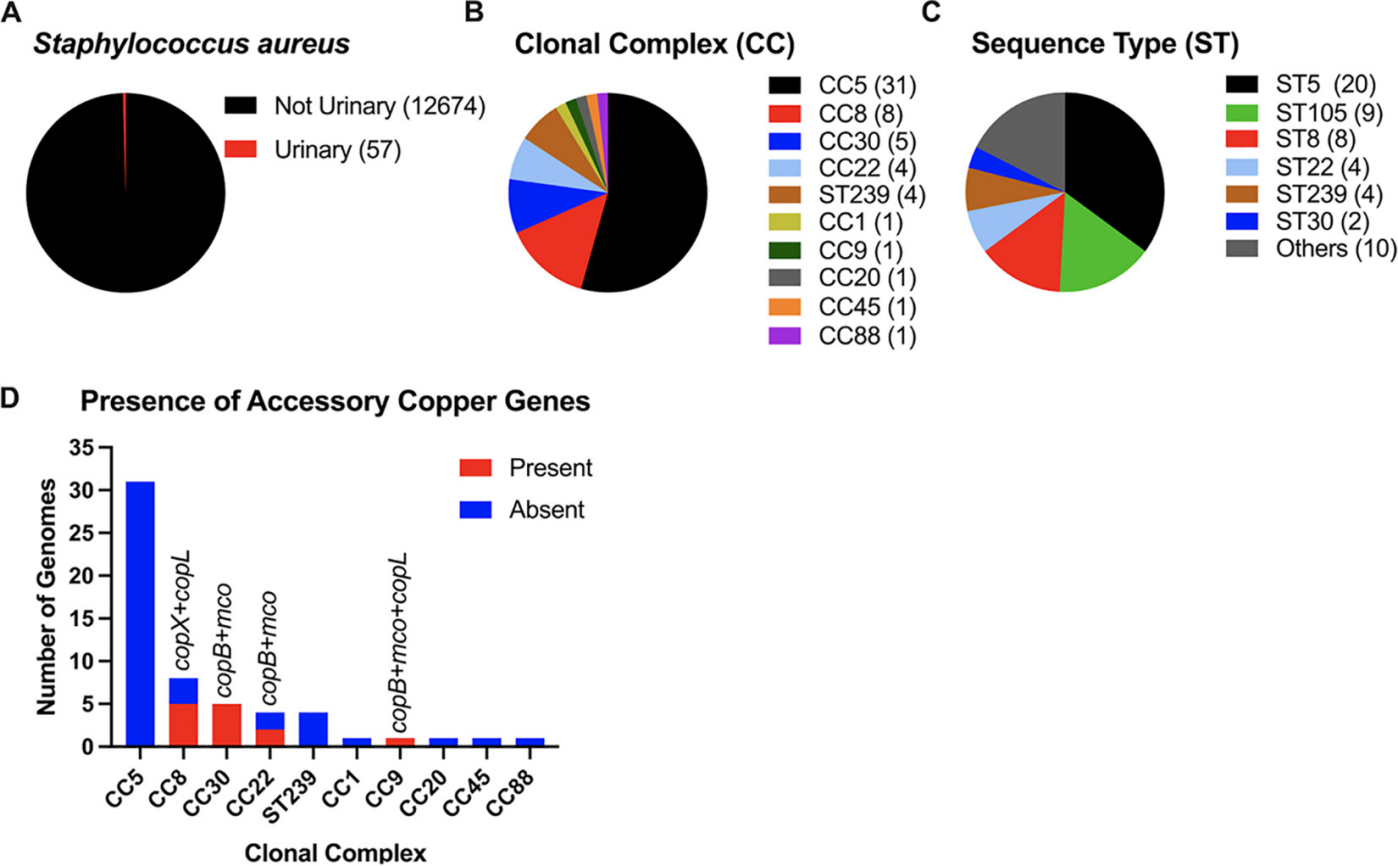
Presence of accessory copper genes in urinary isolates of S. aureus. (A) Pie chart of S. aureus isolates with urine-related terms in GenBank. Fifty-seven from a total of 12,731 isolates have urine-related terms. (B) Pie chart of the different clonal complexes (CCs) for the 57 urinary S. aureus isolates in panel A. The hybrid lineage ST239 is part of CC8 but noted separately. (C) Pie chart of the different sequence types (STs) for the 57 urinary S. aureus isolates in panel A. Only STs with 2 isolates or more are shown. Ten STs representing 10 isolates (ST1 [CC1], ST9 [CC9], ST34 [CC30], ST36 [CC30], ST39 [CC30], ST45 [CC45], ST88 [CC88], ST231 [CC5], ST513 [CC20], and ST632 [CC5]) were categorized as others for easier visualization. (D) Bar plot of presence of copper genes (*copX* [ABX28125.1], *copL* [ABX28126.1], *mco* [WP_019169201.1], and *copB* [WP_000069452.1]) in the 57 urinary S. aureus isolates shown in panel A.

Clinical isolates, particularly MRSA isolates, are known to carry *copX*, *copL*, *copB*, and/or *mco* genes that confer increased copper resistance compared to strains that contain only the conserved copper resistance gene *copA* (20, 21, 28, 30). We probed the publicly available genomes of S. aureus and identified 57 urine-related isolates in a collection of 12,731 genomes in GenBank (Fig. 3A and Table S2). *In silico* multilocus sequence typing (MLST) was used to assign these isolates to a clonal complex (CC) and sequence type (ST). Isolates belonging to CC5, CC8, CC30, ST5, ST105, and ST8 were overrepresented in urine-related S. aureus isolates (Fig. 3B and C). However, this likely reflects overrepresentation of these lineages overall in the database. Out of 57 urine isolates, 13 (22.8%) had two or more accessory copper resistance genes (Fig. 3D). MRSA strains from the USA300 lineage (FPR3757/SA116 and SF8300; Table 1) were used in this study because they belong to ST8, are similar to the well-characterized urine MRSA isolates (23, 36, 37), and are highly prevalent (38).

10.1128/mBio.02038-21.6 TABLE S2 Oligonucleotide primers used in this study. Download Table S2, DOCX file, 0.01 MB. Copyright © 2021 Saenkham-Huntsinger et al. 2021 Saenkham-Huntsinger et al. https://creativecommons.org/licenses/by/4.0/ This content is distributed under the terms of the Creative Commons Attribution 4.0 International license.

### A mouse model of UTI caused by S. aureus.

**FIG 4:**
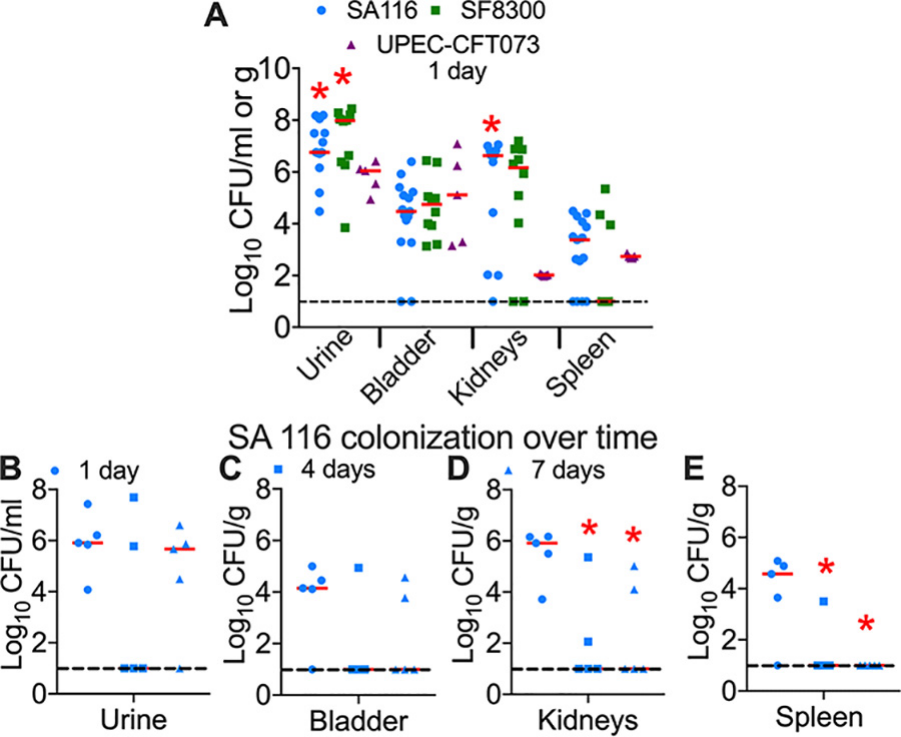
S. aureus colonization in the urinary tract of female CBA/J mice. Urine and tissue bacterial load in mice (*n* = 5 to 15/group) inoculated with MRSA strains SA116 and SF8300 and uropathogenic E. coli (UPEC) strain CFT073. Urine and organs were collected for plate counts. *, *P < *0.05 by Mann-Whitney test compared to CFT073. Symbol, data from a mouse; bars, median; dotted line, limit of detection.

**FIG 5:**
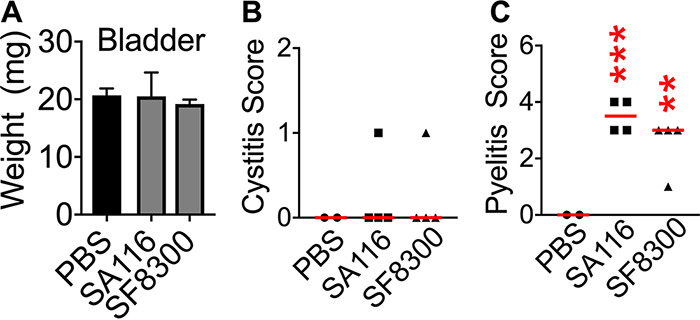
S. aureus induces inflammation in the renal pelvis. (A) Weight of urinary bladders at 24 h postinoculation. Means and SEM are reported. (B) Hematoxylin- and eosin-stained bladder sections were evaluated for tissue damage and inflammation. (C) Kidney sections were also evaluated for tissue damage and inflammation. *n* = 2 to 4/group, and bars indicate medians. **, *P < *0.01; ***, *P < *0.001, both by Mann-Whitney test compared to PBS controls.

As a first step to investigate the role of copper at the host-pathogen interface in the urinary tract, we asked if S. aureus could colonize and infect a murine model of ascending, uncomplicated UTI. Adult female CBA/J mice were inoculated with MRSA strains SA116 and SF8300 (Table 1). Bacterial burden in the urine, urinary bladder, kidneys, and spleens were determined at 24 h postinoculation. Both strains of S. aureus successfully colonized the murine urinary tract (Fig. 4A). Urine bacterial load was higher in S. aureus groups than UPEC strain CFT073 (∼10^7^ versus 10^6^ CFU/ml; Fig. 4A). S. aureus colonization was comparable to that of UPEC in the bladders (∼10^5^ CFU/g) and superior to that of UPEC in the kidneys (∼10^6^ versus 10^2^ CFU/ml; Fig. 4A). Consistent with increased colonization in the kidneys, experimental UTI with S. aureus led to bacteremia, as indicated by colonization of the spleen (Fig. 4A). We then compared colonization of MRSA strain SA116 in the urinary tract up to 7 days postinoculation (dpi) (Fig. 4B to E). Urine had comparable bacterial loads at 1 and 7 dpi but was variable at 4 dpi (Fig. 4B). The highest bacterial burden was observed at 1 dpi in bladders, kidneys, and spleens, with lower loads at later time points (Fig. 4C to E). Compared to sham-inoculated mice, there was no difference in the weights of bladder, suggesting a lack of robust inflammation in response to the presence of S. aureus at 1 dpi (Fig. 5A). Histopathological evaluation of bladder sections revealed no remarkable differences at 1 dpi between control and S. aureus groups (Fig. 5B). A significant induction of inflammation of the renal pelvis (pyelitis) in the kidneys was observed in S. aureus-infected mice compared to controls at 1 dpi (Fig. 5C). Levels of myeloperoxidase, a marker for neutrophil presence, and proinflammatory cytokines (interleukin-1 beta [IL-1β], IL-6, and tumor necrosis factor alpha [ΤΝF-α]) were not significantly different between control and S. aureus groups in bladders and urine at 1 dpi (Fig. S2A to E]. Kidneys from mice infected with S. aureus strain SA116 had elevated levels of IL-1β and IL-6 at 1 dpi compared to phosphate-buffered saline (PBS) controls (Fig. S2C and D).

10.1128/mBio.02038-21.2 FIG S2 Markers of inflammation in urine and organs of the urinary tract. Download FIG S2, DOCX file, 0.06 MB. Copyright © 2021 Saenkham-Huntsinger et al. 2021 Saenkham-Huntsinger et al. https://creativecommons.org/licenses/by/4.0/ This content is distributed under the terms of the Creative Commons Attribution 4.0 International license.

### Sensitivity of S. aureus to excess copper and iron starvation.

**FIG 6:**
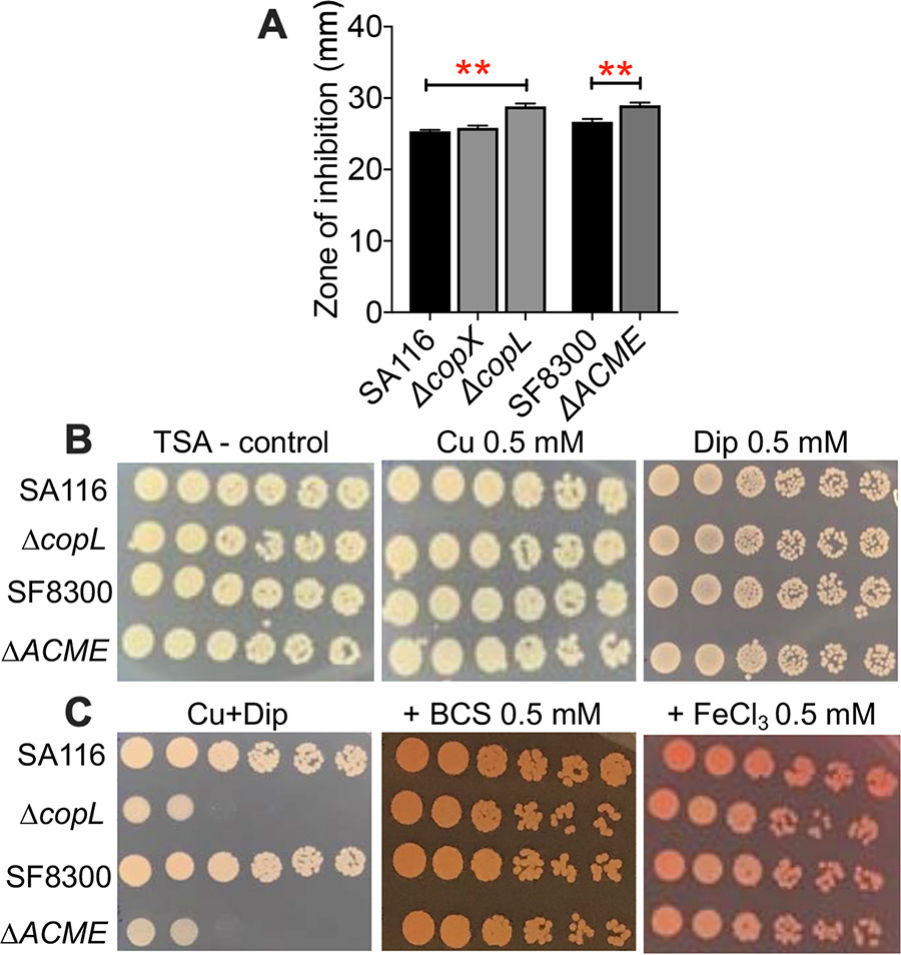
Copper sensitivity of S. aureus is exacerbated by iron starvation. (A) Zones of inhibition of growth of S. aureus wild-type (SA116 and SF8300) and Cu-sensitive mutants (lacking *copX*, *copL*, and ACME) by copper (Cu, 2 M). **, *P < *0.01, ANOVA with Dunnett’s post test. Means and SEM from three independent experiments are reported. (B) Growth of MRSA strains at sublethal levels of Cu and dipyridyl (Dip, a ferric iron chelator). (C) Growth of S. aureus wild-type and Cu-sensitive mutants exposed to Cu excess and iron starvation. Bathocuproine (BCS) was used to chelate Cu, and ferric chloride (FeCl_3_) was used to supplement iron. All assays were repeated at least three times, and a representative image is depicted here.

**FIG 7:**
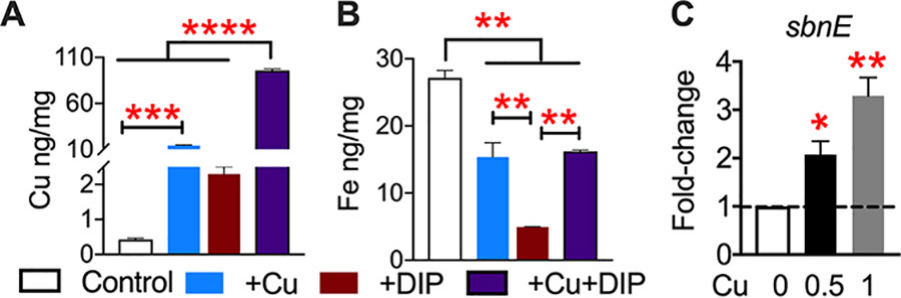
Cellular metal content during copper stress. Cell-associated levels of copper (A) and iron (B) in MRSA strain SA116 exposed to control, copper, dipyridyl, and both, as determined by ICP-MS. Copper and iron content are normalized to the weight of cell pellets. (C) Fold change in abundance of *sbnE* transcript in SA116 in the presence of 0.5 and 1 mM copper sulfate compared to control. Means and SEM from three independent experiments are reported. Dotted line, no change in relative expression. *, *P < *0.05; **, *P < *0.01; ***, *P < *0.001; ****, *P < *0.0001, all by ANOVA with Tukey’s multiple-comparison test.

Concurrent intoxication with copper and starvation for iron is part of the innate immune response against pathogens. First, we verified copper sensitivity phenotypes of MRSA strains SA116 and SF8300 and their SA116Δ*copX*, SA116Δ*copL*, and SF8300ΔACME isogenic mutants. Mutants lacking *copL* and ACME exhibited marked increases in sensitivity to copper, reflected by larger zones of inhibition than their parental strains (Fig. 6A). The *copX* mutant had a slightly larger zone of inhibition than the wild-type strain, but this difference was not statistically significant (Fig. 6A). These mutants exhibited wild-type levels of survival when subjected to iron starvation imposed by supplementation with dipyridyl, a chelator of ferric iron (Fig. 6B and Fig. S3A). However, sensitivity to copper in the mutant strains was exacerbated during iron limitation (Fig. 6C). At higher concentrations of copper and dipyridyl (1 mM each), growth of wild-type strains was also abrogated (Fig. S3A). Importantly, chelation of copper with bathocuproine or addition of iron rescues S. aureus from lethal stress induced by combined copper excess and iron starvation (Fig. 6C and Fig. S3B). Copper chelation or iron supplementation, up to 1 mM concentration used in our assays, does not affect the viability of these strains (Fig. S3C and D). To further investigate the connection between homeostasis of copper and iron, we determined the levels of cell-associated copper and iron during copper stress and iron starvation in MRSA strain SA116 by ICP-MS (Fig. 7). As anticipated, copper stress resulted in an increase in the cellular level of copper (Fig. 7A). MRSA supplemented with both copper and dipyridyl exhibited a significant increase in the accumulation of copper compared to control, copper, or dipyridyl treatment groups (Fig. 7A). Conversely, addition of copper resulted in decreased levels of cellular iron, and dipyridyl was used as a positive control in this assay (Fig. 7B). Dipyridyl was less effective at depleting cellular iron levels in MRSA exposed to copper (Fig. 7B), suggesting an interaction between copper and dipyridyl. To test whether bioavailability of iron is diminished during copper stress, we determined the expression of *sbnE* involved in the biosynthesis of staphyloferrin B, a siderophore. Real-time PCR experiments revealed that exposure to copper led to an increase in the abundance of *sbnE* transcripts compared to controls (Fig. 7C). Collectively, these data establish ACME and *copL* as critical components in resistance to copper and reveal a connection between copper toxicity and iron homeostasis in S. aureus.

10.1128/mBio.02038-21.3 FIG S3 Copper sensitivity is linked to iron homeostasis in S. aureus. Download FIG S3, DOCX file, 0.7 MB. Copyright © 2021 Saenkham-Huntsinger et al. 2021 Saenkham-Huntsinger et al. https://creativecommons.org/licenses/by/4.0/ This content is distributed under the terms of the Creative Commons Attribution 4.0 International license.

### ACME and copper resistance gene *copL* promote fitness of S. aureus during UTI.

**FIG 8:**
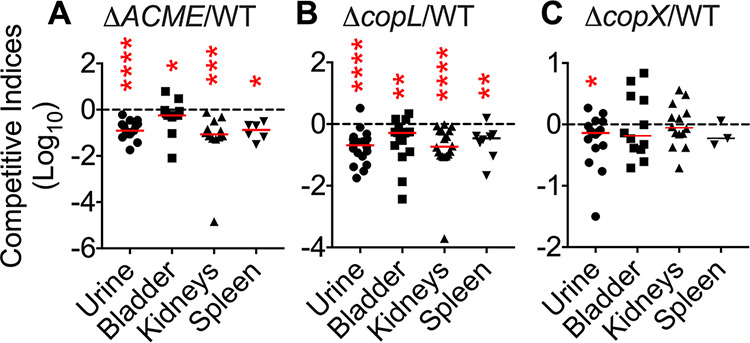
ACME and *copL* contribute to pathogen fitness during UTI. Mice (female, CBA/J, *n* = 10/group) were inoculated with a mixture of wild-type and mutant strains. Urine and organs were collected at 24 h postinoculation. Competitive indices were calculated as the ratio of mutant to wild type *in vivo* and were normalized to the inoculum. (A) Competitive indices for the ACME mutant compared to SF8300. (B) Competitive indices for the *copL* mutant compared to SA116. (C) Competitive indices for the *copX* mutant compared to SA116. Dotted line, no loss of fitness in the mutant compared to the wild-type strain (competitive index = 1). Each symbol corresponds to results from a mouse, and the median is indicated with bars. *, *P < *0.05; **, *P < *0.01; ***, *P < *0.001; ****, *P < *0.0001, all by Wilcoxon signed-rank test.

First, we assessed the potential of wild-type and mutant strains to grow in human urine to mimic the milieu encountered during infection. There was no significant difference in the growth rate (Fig. S4A) or final biomass (Fig. S4B) between these strains when cultured in urine from healthy volunteers and when urine was supplemented with copper found in UTI urine samples (Fig. S4C and D). We tested the role of ACME and accessory copper resistance genes *copX* and *copL* in the survival and growth of S. aureus in the urinary tract. Coinfection experiments were performed with a mixture containing equal numbers of wild-type and isogenic mutants, and bacterial load was enumerated in urine and tissues after 24 h postinoculation. Lack of ACME was associated with a significant decrease in fitness of the mutants in urine (median competitive index of 0.1), bladder (0.5), kidneys (0.09), and spleen (0.1) relative to its parental wild-type strain, SF8300 (Fig. 8A). The mutant lacking *copL* exhibited decreased fitness in urine (0.2), bladder (0.5), kidneys (0.1), and spleen (0.3) relative to its parental wild-type strain, SA116 (Fig. 8B). Decreased fitness for these mutants was most pronounced in urine and kidneys, followed by spleen and urinary bladder (Fig. 8A and B). Competitive indices for the *copL* mutant were lower than that of the *copX* mutant (Fig. 8A to C). The mutant lacking *copX* did not exhibit any change in fitness (Fig. 8C) relative to the parental strain, SA116.

10.1128/mBio.02038-21.4 FIG S4 Growth of S. aureus in human urine. Download FIG S4, DOCX file, 0.3 MB. Copyright © 2021 Saenkham-Huntsinger et al. 2021 Saenkham-Huntsinger et al. https://creativecommons.org/licenses/by/4.0/ This content is distributed under the terms of the Creative Commons Attribution 4.0 International license.

### Copper supplementation impacts S. aureus colonization in the murine urinary tract.

**FIG 9:**
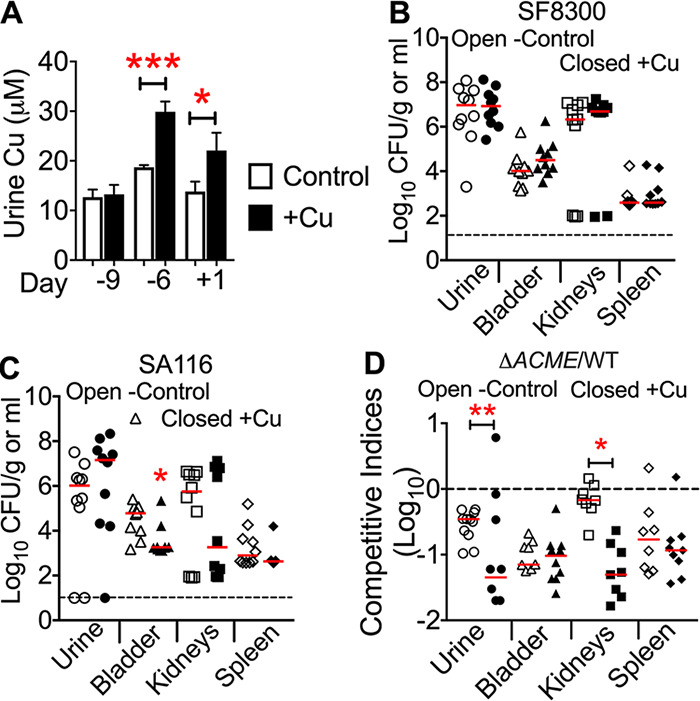
Impact of urinary copper levels of S. aureus colonization. (A) Mice (female CBA/J, *n* = 10/group) were provided water with or without copper (Cu) supplementation (0.5 g/liter). Urine copper levels were determined by ICP-MS at the indicated days. Means and SEM are reported. *, *P < *0.05; ***, *P < *0.001, both by ANOVA with Dunnett’s posttest. Mice receiving control or copper-supplemented water were inoculated with wild-type strain SF8300 (B) or SA116 (C), and bacterial burden was determined. (D) Coinfection of SF8300 and ACME mutant was conducted in mice receiving control or copper-supplemented water, and competitive indices were calculated. *, *P < *0.05; **, *P < *0.001, Mann-Whitney test compared to controls.

First, we tested whether increased levels of copper in the urine would alter bacterial load during UTI. Copper-supplemented water was administered for 9 days prior to induction of UTI with wild-type USA300 MRSA strains SF8300 and SA116 or coinfection with SF8300 and its isogenic ACME mutant. Copper-supplemented mice had significantly higher levels of urinary copper than pretreatment samples (day −9) and the control group (Fig. 9A). There was no difference in colonization of wild-type SF8300 strain in the urinary tract between copper-supplemented and control mice (Fig. 9B). Tissue bacterial load of wild-type SA116 was significantly decreased in the urinary bladders of copper-supplemented mice (∼10^3^ CFU/g) compared to controls (∼10^5^ CFU/g; Fig. 9C). Although there was a 2 orders of magnitude decrease in the median kidney bacterial load in copper-supplemented mice, this difference was not statistically significant (Fig. 9C). Since there was no copper-dependent change in colonization by strain SF8300, we conducted coinfection experiments with the ACME mutant and SF8300 in control and copper-supplemented mice. If ACME-mediated enhanced copper resistance plays a role in fitness in the urinary tract, then an ACME mutant would be anticipated to have a lower competitive index (decreased fitness) in copper-supplemented mice compared to controls. The coinfection experiment revealed that the competitive indices of the ACME mutant in urine and kidneys were significantly lower in the copper-supplemented mice than control mice (Fig. 9D). There was no difference in the competitive indices in bladder and spleen from control and treatment groups (Fig. 9D). Taken together, these findings suggest that enhanced copper resistance confers a fitness advantage for S. aureus during colonization of the murine urinary tract.

## DISCUSSION

Here, we report that copper and ceruloplasmin are mobilized to urine as part of the host response activated during UTI caused by Gram-positive pathogens. Urinary copper and ceruloplasmin content are elevated during UTI caused by UPEC and other Gram-negative pathogens (32–34, 39). Importantly, experimental UPEC-induced UTI in a nonhuman primate model triggered urinary copper mobilization, demonstrating a cause-effect relationship between UTI and urinary copper mobilization (32). Taken together with our current findings on UTI caused by Gram-positive pathogens, copper and ceruloplasmin emerge as conserved host effectors responding to bacterial colonization in the urinary tract. Although there is an increase in urinary copper content during UTI compared to healthy controls, levels of copper present in the urine are not sufficient for direct bactericidal activity. Toxic levels of copper (up to ∼0.5 mM) have been reported to accumulate within the phagosomes of macrophages containing engulfed pathogens (40). Murine macrophages have been demonstrated to utilize copper to kill a laboratory strain of E. coli (41). Directly relevant for this study is the finding that horizontally transferred copper resistance genes (*copB*, *copX*, and *copL*) in MRSA confer enhanced resistance to killing within macrophages and in human blood (28, 30). We posit that increases in urinary copper content during UTI facilitate more effective killing of pathogens by macrophages.

MRSA strains carry horizontally transferred genes involved in copper resistance, in addition to the *copA* and *copZ* genes found in the core genome of S. aureus (25, 28–30). Both North American and South American clones of MRSA isolates derived from USA300 lineage carry the ancillary copper resistance system with *copX* and *copL* genes (21), which are also found in the genomes of urine-related S. aureus isolates. Members of the USA300 MRSA lineage are more pathogenic, globally distributed, and known for being successfully transmitted in community settings (20, 38). The ancillary copper resistance genes have been proposed to confer fitness advantage during infection, bolstered by the observations that these genes promote better survival of S. aureus within RAW 264.7 murine macrophages and in human blood (28, 30). Increased resistance to killing within alveolar macrophages is linked to higher virulence during pneumonia in a mouse model caused by MRSA (42). The role of copper in killing MRSA by alveolar macrophages, however, remains to be addressed experimentally. Here, we provide direct evidence for the role of *copL* in augmenting S. aureus fitness in a murine model of UTI. Interestingly, loss of *copX* does not alter pathogen fitness in our infection model. This finding could be explained by the redundant function of CopA and CopX as copper efflux ATPases (31) and is consistent with our *in vitro* assays where the *copX* mutant exhibits slight, but not statistically significant, increases in resistance to copper compared to the wild-type strain. Purves et al. have reported that the *copX* mutant exhibits significant increases in copper sensitivity when cultured in brain heart infusion or RPMI broth (30). In this study, we determined the zones of growth inhibition on tryptic soy agar. Differences in the growth milieu is the likely reason for differences in the copper sensitivity phenotype of the *copX* mutant.

Loss of copper efflux and detoxification systems in a diverse set of bacterial pathogens, including K. pneumoniae, Listeria monocytogenes, Mycobacterium tuberculosis, Salmonella enterica serovar Typhimurium, Streptococcus pneumoniae, P. aeruginosa, and UPEC, leads to attenuation during infection in animal models (33, 43–49). Our findings reveal a role for copper resistance conferred by CopL, a cell surface copper-binding lipoprotein (31), in S. aureus fitness during UTI. Loss of the ACME genomic region also leads to decreased fitness during UTI, similar to the findings on the role of ACME as a fitness factor in a rabbit model of bacteremia with MRSA (50). ACME encodes multiple genes, including those involved in survival under acid stress and polyamine excess, in addition to *copX* and *copL* (21, 51–53). Loss of fitness of the ACME mutant in the mouse urinary tract raises interesting questions on the relative contribution of functions, including increased copper resistance, survival under acid stress, and tolerance to polyamine excess endowed by ACME in the pathogenesis of UTI caused by MRSA. This report is significant because it sheds light on the role of a gene involved in copper resistance in S. aureus fitness in an animal model of human disease. Previous studies have demonstrated that the transcription of *copA* is induced in S. aureus in a murine renal abscess model (54) and during interaction with human neutrophils (55). Our findings, taken in light of the previous reports, support a model in which copper is an effector of innate immunity utilized in response against infection caused by a wide range of bacterial pathogens infecting various organs within a mammalian host.

Our work has unraveled an unexpected finding that links copper toxicity with iron homeostasis in S. aureus. Toxicity of copper is exacerbated during iron starvation, and this phenotype is reversed upon either iron supplementation or chelation of copper in S. aureus. Determination of cell-associated copper and iron content by ICP-MS indicates a potential copper-ionophore activity, in addition to iron chelation, for dipyridyl in S. aureus. These results, taken in light of the increased copper sensitivity of mutants during concurrent exposure to dipyridyl, further support the proposed roles for these genes in copper resistance in S. aureus. Transcriptome analysis of copper-stressed E. coli revealed increased transcription of genes involved in iron uptake (56). Copper stress induces the biosynthesis of enterobactin, the primary siderophore involved in iron acquisition in E. coli (57). On the contrary, transcriptional profiling of S. aureus during copper stress did not reveal an upregulation of genes involved in iron uptake (26), leading to the conclusion that copper and iron homeostasis mechanisms are not linked in this pathogen. We are interested in the combined effects of copper stress and iron starvation because uropathogens are concomitantly exposed to these conditions in the context of an inflamed urinary tract during UTI (32–34, 58, 59). Our observation on the connection between iron starvation and copper toxicity is consistent with the current paradigm of enzymes containing iron-sulfur clusters as principal targets of copper intoxication in E. coli (60, 61). Since copper is placed at the top of the Irving-Williams series, it has the highest propensity to mismetallate binding sites for other transition metals, such as iron and manganese (62). Our findings demonstrate that homeostatic mechanisms governing copper and iron are interlinked in S. aureus, comparable to the findings previously limited to Gram-negative bacteria (E. coli and P. aeruginosa) (56, 63).

The CBA/J strain of mice develop ascending UTI that includes robust colonization of the lower (bladder) and upper (kidneys) parts of the urinary tract and systemic dissemination (in a subset of mice) following instillation of a uropathogen in the bladder. This model has been used to investigate the bacterial virulence, fitness mechanisms, and host response to UTI (64). Here, we adapted the CBA/J mouse model to investigate whether MRSA strains could colonize and establish UTI in a mammalian host. Our results reveal robust colonization in the urinary tract and also systemic dissemination to the spleen arising from the urinary tract at early stages of UTI (1 dpi). Consistent and robust colonization observed at 1 dpi is lost at 4 and 7 dpi, indicating that MRSA is not an effective colonizer of an otherwise healthy urinary tract. These findings are in accordance with a previous study on a catheter implant-based model of MRSA UTI, despite the differences in MRSA and mouse strains used in these studies (23). Notwithstanding robust colonization in the bladder, MRSA does not induce cystitis in this model. However, extensive pyelitis is triggered by MRSA upon ascension to kidneys and is consistent with kidneys as the primary niche for S. aureus colonization after inoculation by the intravenous route. It is also consistent with high levels of bacteremia (>10%) associated with S. aureus urinary tract infection reported by Muder et al. (12). This model was able to distinguish changes in the relative fitness between wild-type strains and mutants (*ACME-copL*) to survive and proliferate in the urinary tract. Walker et al. have published a pioneering study on how catheter implantation in the mouse urinary bladder alters the niche to augment the virulence of MRSA (23). Their findings demonstrate a role for implant-induced mobilization of fibrinogen to the urinary tract that is subsequently exploited by MRSA for effective adherence and colonization (23). Specifically, loss of clumping factor B (ClfB) in MRSA resulted in decreased colonization of the catheter implant in the C57BL/6 mouse model of community-acquired UTI. Since MRSA causes more UTI in patients with catheter use than in otherwise healthy individuals, an important follow-up study is to compare our findings from the uncomplicated UTI model to the catheter implant model of UTI.

This study establishes a role for copper at the host-pathogen interface during UTI and raises several critical questions to guide future investigations. Our findings raise the question of the extent to which CopA and CopZ found in all strains of S. aureus are involved in pathogen fitness during UTI. Involvement of copper resistance in the fitness of *S. saprophyticus* and S. epidermidis during UTI should be examined to assess if resistance to copper is a shared pathway exploited by staphylococci for survival and growth within hosts. Recently, amino acids and other effectors secreted by P. aeruginosa have been shown to protect S. aureus from copper toxicity (65). Given the common incidence of UTIs of polymicrobial etiology during catheter use, the nexus between bacterial copper resistance and fitness during UTI should also be evaluated in murine models of polymicrobial UTI.

A limitation of this study is that we have not demonstrated that genetic complementation of *copL* restores wild-type levels of fitness for the *copL* mutant in the mouse model. Other groups have demonstrated that the copper sensitivity phenotype could be fully complemented *in vitro* by reintroducing *copL* (30, 31). Our *in vitro* assays, including growth in human urine, have not revealed a fitness defect for this mutant in the absence of copper stress, lending confidence to our finding that the loss of fitness phenotype in the host is specifically caused by lack of copper sequestration by CopL. In summary, our investigation reveals that copper and ceruloplasmin are mobilized to urine during UTI caused by Gram-positive uropathogens. Copper-sensitive mutants lacking the ACME genomic region and *copL* exhibit decreased fitness during UTI in a murine model. We demonstrate that copper intoxication is linked to disruption of iron homeostasis in S. aureus. Collectively, our findings suggest a protective role for copper against pathogen colonization in the urinary tract.

## MATERIALS AND METHODS

### Human urine samples.

Deidentified urine samples submitted with ICD-10 code N39.0 (UTI, site not specified) were collected from the clinical microbiology laboratory at the Wake Forest Baptist Medical Center in accordance with an IRB-approved protocol (IRB00035856). Urine samples from healthy volunteers were also collected according to protocol IRB00035856. Urine samples were analyzed using dipsticks (Fisher), and UTI urine samples positive for leukocyte esterase and negative for nitrite and erythrocytes were used in this study. Samples were cultured on MacConkey, lysogeny, tryptic soy, and Mueller-Hinton agar. Mass spectrometry was used to determine the identity of urine isolates. We used urine samples that contained S. aureus (*n* = 6), S. epidermidis (*n* = 3), *S. saprophyticus* (*n* = 2), *S. haemolyticus* (*n* = 6), *S. cohnii* (*n* = 1), S. hominis (*n* = 1), *S. condimenti* (*n* = 1), and E. faecalis (*n* = 20) and a polymicrobial (*n* =12) containing two or more of the following organisms: Acinetobacter nosocomialis, Arthrobacter cumminsii, Corynebacterium minutissimum, E. faecalis, *S. haemolyticus*, and *S. condimenti.* Cell-free urine samples were obtained by centrifugation to remove to host cells and tissue debris. Creatinine content was determined using a colorimetric assay (Cayman Chemicals). A refractometer (Sper Scientific) was used to determine the specific gravity of urine samples.

### ICP-MS.

Urine (whole or cell-free human and mouse) and bacterial pellets were digested with trace metal-grade nitric acid and diluted with trace metal-grade water before introduction into an ICP-MS instrument (8800 ICP-MS; Agilent Technologies). All samples were analyzed in triplicate, with a limit of detection of 0.3 nM copper, as reported previously (32). Copper was determined at *m/z* 63, in single quadrupole mode, using He (3.5 ml/min) in the ICP-MS collision/reaction cell to minimize spectral interferences.

### Ceruloplasmin detection.

ELISA was performed on urine samples in duplicate according to the manufacturer’s instructions (Molecular Innovations). Bicinchoninic acid assay revealed that these urine samples had comparable levels of total protein content. Immunoblotting was performed on urine samples (10 μl) resolved in 10% SDS-PAGE, transferred to polyvinylidene difluoride membrane, blocked, and probed with an anti-ceruloplasmin antibody (Thermo Scientific). Anti-rabbit IgG-horseradish peroxidase conjugate was used for detection by chemiluminescence (ECL Prime; Amersham). Human ceruloplasmin (Athen Research and Technology) was used as a positive control. Images were acquired in a Gel-Doc system (Bio-Rad laboratories) and cropped to display signal indicating holoceruloplasmin (132 kDa). A representative image is depicted in Fig. 2C.

### Ceruloplasmin degradation.

Human ceruloplasmin (0.05 μg/ml, final concentration) was incubated with MRSA strain SA116 or culture supernatant from SA116 at 37°C for 16 h. Immunoblotting was performed, as described above, to detect intact and degraded ceruloplasmin.

### Bioinformatic analysis of S. aureus genomes.

The biosample attributes for all 12,731 Staphylococcus aureus genomes currently available at GenBank (66) were checked for urinary-related terms (urosepsis, urologic, urinary tract, urinary tract infection, UTI, urine, kidney, pyelonephritis, cystitis, ureter, urethra, urinary, urinary catheter, urethritis, and hematuria) using a custom script that uses the Entrez Programming Utilities (https://www.ncbi.nlm.nih.gov/books/NBK25501/) from NCBI. The RefSeq genomes of the 57 urinary related isolates (see Table S1 in the supplemental material) were downloaded using WhatsGNU (67), and sequence types were determined using the MLST tool available at https://github.com/tseemann/mlst. The presence of accessory copper resistance genes (*copX* [ABX28125.1], *copL* [ABX28126.1], *mco* [WP_019169201.1], and *copB* [WP_000069452.1]) was checked using Abricate (https://github.com/tseemann/abricate).

10.1128/mBio.02038-21.5 TABLE S1 Accessory copper resistance genes in the genomes of urine-related S. aureus isolates. Download Table S1, XLSX file, 0.02 MB. Copyright © 2021 Saenkham-Huntsinger et al. 2021 Saenkham-Huntsinger et al. https://creativecommons.org/licenses/by/4.0/ This content is distributed under the terms of the Creative Commons Attribution 4.0 International license.

### Copper sensitivity and iron starvation assays.

S. aureus strains (Table 1) were cultured in tryptic soy broth (TSB) to stationary phase (∼20 h). Cultures were spread on tryptic soy agar (TSA), and a paper disc immersed in 5 μl of 2 M copper sulfate solution was placed in the center. Zones of growth inhibition were measured the next day. Dilutions of overnight cultures were spot plated on TSA containing copper, dipyridyl, bathocuproine, and/or ferric chloride at concentrations indicated in the figure legends (Fig. 6 and Fig. S3). Plates were incubated at 37°C overnight, and images were acquired the following day.

### Cellular metal content.

Overnight cultures of MRSA strain SA116 were diluted and cultured in fresh TSB supplemented with 0.5 mM copper sulfate, 0.5 mM dipyridyl, or both for 90 min. Cells were pelleted and washed with 10 mM HEPES containing 0.5 mM EDTA as reported previously (68). Cell pellets were weighed and processed for ICP-MS analysis as described above.

### Culture in human urine.

S. aureus strains in stationary-phase cultures in tryptic soy broth were washed in PBS and diluted 1:1,000 in 3 ml of filter-sterilized, pooled urine samples from healthy volunteers (Cone Bioproducts). When indicated, urine was supplemented with 0.5 μΜ copper sulfate to simulate elevated copper content of UTI urine samples. The optical density at 600 nm (OD_600_) was recorded after 24 h of incubation at 37°C with shaking at 200 rpm. S. aureus strains cultured to stationary phase in human urine were diluted 1:1,000 into fresh urine, and the OD_600_ was recorded at 30-min intervals over 16 h in a microplate reader (BioTek Instruments).

### Quantitative PCR.

Overnight cultures of MRSA strain SA116 were diluted and cultured in human urine to mid-exponential phase prior to addition of human holoceruloplasmin (100 ng/ml) or copper (0.5 and 1 mM). RNAprotect (Qiagen) was added after 30 min, cells were treated with lysostaphin (Sigma), and RNA was extracted with an RNeasy minikit (Qiagen). DNase treatment (Turbo DNA-free; Ambion), reverse transcription (Superscript III; Invitrogen), and SYBR green-based quantitative PCR in a CFX real-time system (Bio-Rad Laboratories) were performed essentially as described earlier (68). Oligonucleotide primers used in this study are listed in Table S2. Levels of transcripts were normalized to *gyrA*, and relative expression was calculated using the untreated control as the calibrator.

### Murine model of UTI.

Experiments using mice were approved by institutional committees at the Texas A&M University (2018-0362) and Wake Forest Baptist Medical Center (15-178). Female CBA/J mice 4 to 6 weeks age were used, and the numbers of mice used in specific experiments are listed in the figure legends (Fig. 4, 5, 8, and 9). MRSA and UPEC strains were cultured in tryptic soy broth and lysogeny broth, respectively, for infection studies. Mice were anesthetized with tribromoethanol, and ∼10^8^ CFU of S. aureus strain SA116 or SF8300 or uropathogenic E. coli strain CFT073 in 50 μl was instilled in the urinary bladder using a syringe pump to avoid induction of vesicoureteral reflux, as we have reported previously (69). The syringe pump was operated at a flow rate of 100 μl/min to avoid unintentional induction of vesicoureteral reflux and to maximize reproducibility. Urine was collected and animals were euthanized prior to collection of bladder, kidneys, and spleen on day 1, 4, or 7 postinoculation. Organs were homogenized and plate counts were determined. CFU counts were normalized to milliliters and grams for urine and tissue samples, respectively. Median number of CFU/g or milliliter is indicated in Results.

### Histopathology.

Bladders and kidney sections were fixed in formalin prior to embedding in paraffin. Sections were stained with hematoxylin and eosin and evaluated in a blinded manner by a board-certified veterinary anatomic pathologist as described earlier (70). Criteria used for assessment include the presence of neutrophils, degree of inflammatory changes, presence of bacteria, and tissue damage.

### ELISA.

Urine and homogenates of bladder and kidneys were used for determination of myeloperoxidase (MPO; ThermoScientific), IL-1β, IL-6, and TNF-α (R&D Systems). Assays were performed in duplicate and in accordance with the manufacturer’s instructions, as we have reported earlier (70).

### Competitive indices.

Mice were inoculated as described above, except with a 1:1 mixture of wild-type strains and mutants (SF8300 and ΔACME, SA116 and Δ*copL*, and SA116 and Δ*copX* strains). Urine and organs were collected as described above. Plate counts of wild-type and mutant strains were enumerated on TSA and TSA with rifampin. Both wild-type and mutant strains grow on TSA, whereas only the mutant strains grow on TSA with rifampin. Competitive indices were calculated as the ratio of number of CFU of mutant to wild type in urine or tissue, as recently described (68). This was normalized to the ratio of CFU number of mutant to wild type in the inoculum. Median competitive indices are indicated in Results.

### Copper supplementation in drinking water.

Mice were provided water containing copper sulfate (0.5 g/liter) for 9 days prior to and a day after inoculation with S. aureus. Urine samples were collected on days −9, −6, and +1 for determination of copper content by ICP-MS analysis.

### Statistical analyses.

Data were analyzed in GraphPad Prism v7 with appropriate tests indicated in the figure legends. A *P *value of <0.05 or lower was considered a statistically significant difference.
