# Supplementary material for: Copper Resistance Promotes Fitness of Methicillin-Resistant Staphylococcus aureus during Urinary Tract Infection
Source: mBio. 2021 Sep 7;12(5):e02038-21. doi: 10.1128/mBio.02038-21 (PMC8546587; doi:10.1128/mBio.02038-21)
Supplement: FIG S1 [file mbio.02038-21-sf001.docx]

**
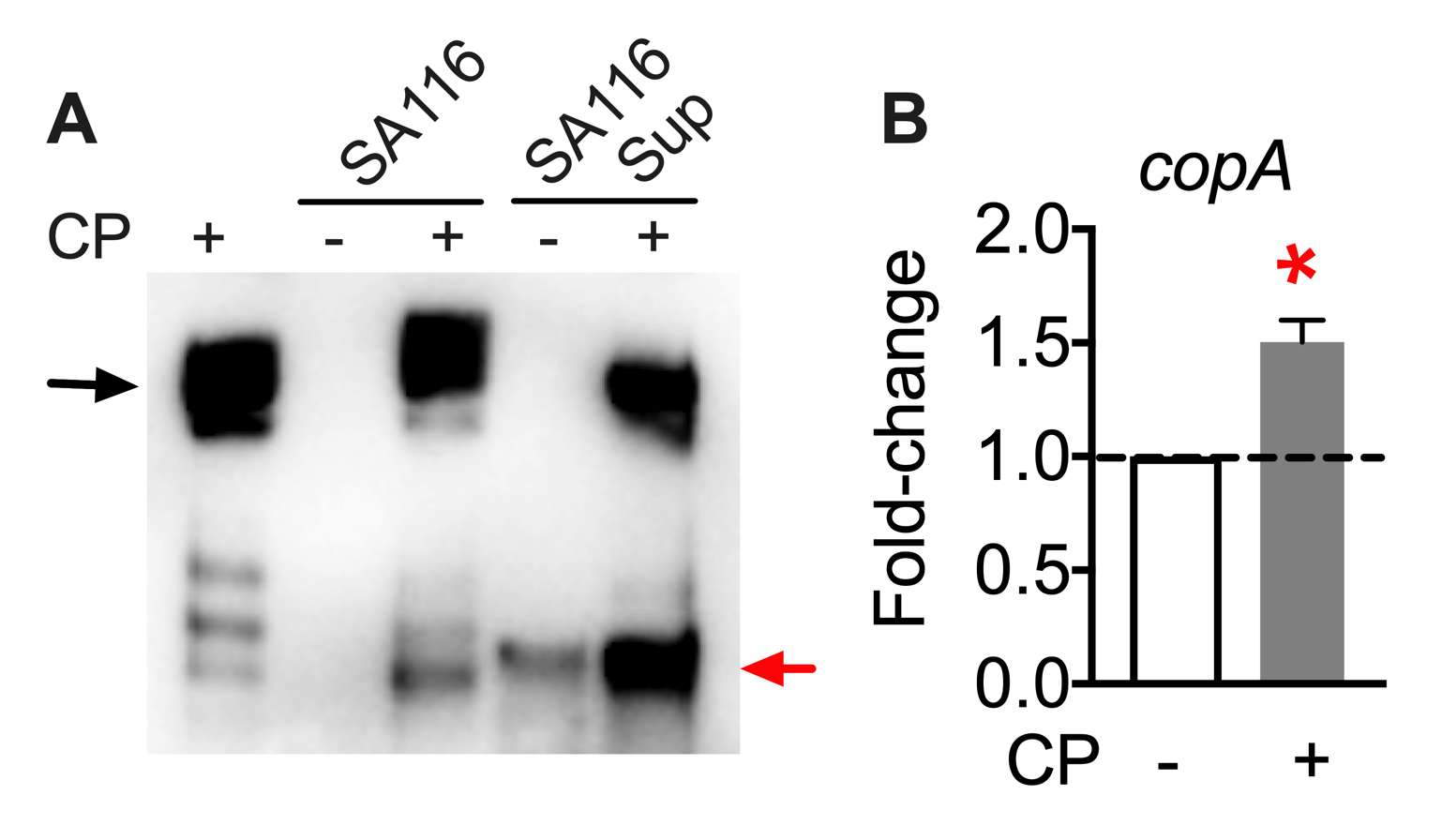
**

**Figure S1. MRSA-ceruloplasmin interaction assays.** (A) Incubation of pure human holoceruloplasmin (50 ng/μl, black arrow) with MRSA strain SA116 leads to degradation (red arrow). MRSA culture supernatant has strong ceruloplasmin degrading activity (red arrow). A representative blot from three independent experiments is depicted here. (B) Expression of *copA* gene in SA116 exposed to ceruloplasmin (100 ng/ml, comparable to UTI urine), relative to control. Mean and SEM from three independent experiments are presented. **P* < 0.05, *t*-test.
