# Supplementary material for: Copper Resistance Promotes Fitness of Methicillin-Resistant Staphylococcus aureus during Urinary Tract Infection
Source: mBio. 2021 Sep 7;12(5):e02038-21. doi: 10.1128/mBio.02038-21 (PMC8546587; doi:10.1128/mBio.02038-21)
Supplement: TABLE S2 [file mbio.02038-21-st002.docx]

**Table S2. Oligonucleotide primers used in this study**

| **Gene** | **Orientation^a^** | **Sequence** |
| --- | --- | --- |
| *gyrA* | F | TTGTTAGAAGACGTACGCAA |
| *gyrA* | R | GTGCGATACGTAATCCTTCT |
| *copA* | F | TAAGAGTGCAAGTGGGTTTT |
| *copA* | R | ACATCTTGATGCACAGCTAA |
| *sbnE* | F | TAAATAAAATGGACGGCGTTC |
| *sbnE* | R | AATGCGAGTGACAAGTTTATCAG |

^a^F, forward; R, reverse
