# Supplementary material for: Copper Resistance Promotes Fitness of Methicillin-Resistant Staphylococcus aureus during Urinary Tract Infection
Source: mBio. 2021 Sep 7;12(5):e02038-21. doi: 10.1128/mBio.02038-21 (PMC8546587; doi:10.1128/mBio.02038-21)
Supplement: FIG S2 [file mbio.02038-21-sf002.docx]

**Figure S2. Markers of inflammation in urine and organs of the urinary tract.** ELISA was used to determine the levels of myeloperoxidase (MPO, A), TNF-α (B), IL-6 (C), and IL-1β (D) in the urinary bladder and kidneys at 24 hours post-inoculation with wild-type *S. aureus* strains SA116 and SF8300. MPO levels were also determined in urine (E). Mean and SEM are reported. **P* < 0.05, ANOVA with Dunnett’s post-test, compared to PBS controls.
