# Supplementary material for: Copper Resistance Promotes Fitness of Methicillin-Resistant Staphylococcus aureus during Urinary Tract Infection
Source: mBio. 2021 Sep 7;12(5):e02038-21. doi: 10.1128/mBio.02038-21 (PMC8546587; doi:10.1128/mBio.02038-21)
Supplement: FIG S3 [file mbio.02038-21-sf003.docx]

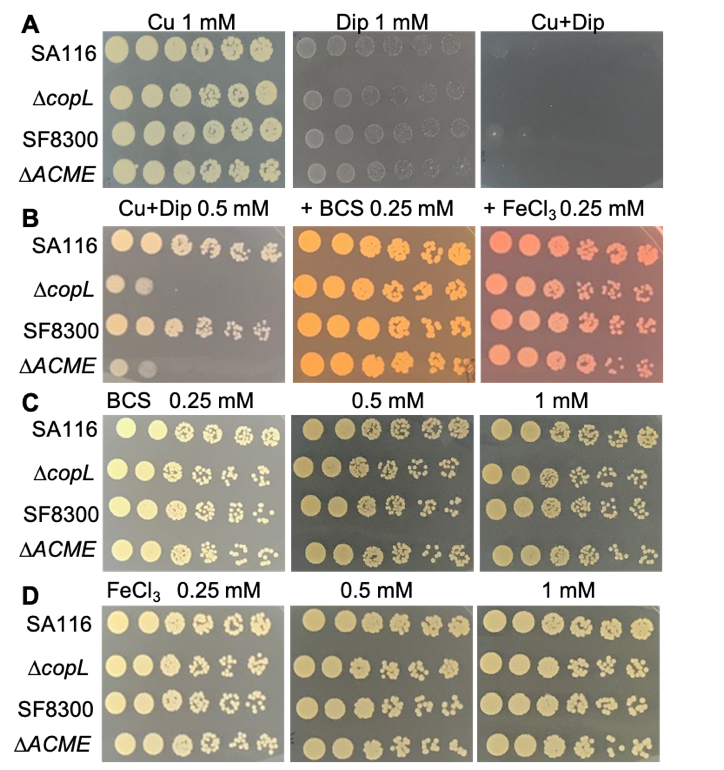


**Figure S3. Copper sensitivity is linked to iron homeostasis in *S. aureus***. Serial dilutions of wild-type and copper-sensitive mutant strains were plated on TSA containing indicated concentrations of copper (Cu), dipyridyl (Dip), bathocuproine (BCS), and/or ferric chloride (FeCl_3_). Plates were incubated for 24 hours at 37°C prior to imaging. All assays were repeated at least three times, and a representative image is depicted here.
