# Supplementary material for: Copper Resistance Promotes Fitness of Methicillin-Resistant Staphylococcus aureus during Urinary Tract Infection
Source: mBio. 2021 Sep 7;12(5):e02038-21. doi: 10.1128/mBio.02038-21 (PMC8546587; doi:10.1128/mBio.02038-21)
Supplement: FIG S4 [file mbio.02038-21-sf004.docx]

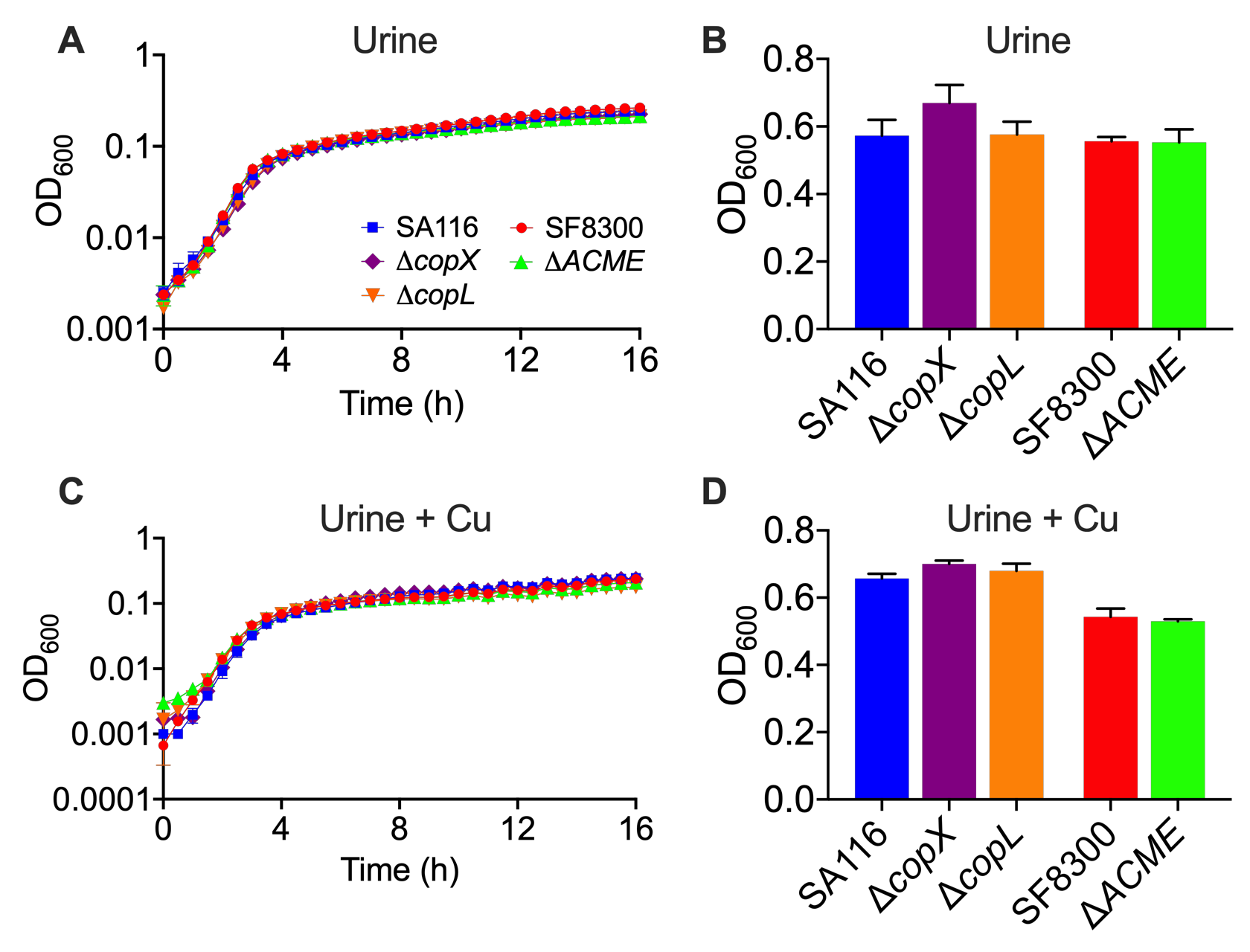


**Figure S4. Growth of *S. aureus* in human urine.** (A&B) *S. aureus* strains were inoculated in filter-sterilized urine from healthy adult volunteers. (A) Optical density of 96 well plates, incubated at 37°C, was recorded at 30 minute intervals for 16 hours. (B) Optical density values of cultures in human urine after 16 hours of growth in culture tubes. (C&D) *S. aureus* strains were inoculated in filter-sterilized urine from healthy adult volunteers supplemented with 0.5 μM copper sulfate. Kinetic (C) and end-point (D) reads of optical density were collected as in A&B. Mean and SEM are presented. Error bars for some data points are too small to be seen.
